# Development of orphan vaccines: an industry perspective.

**DOI:** 10.3201/eid0506.990602

**Published:** 1999

**Authors:** J Lang, S C Wood

**Affiliations:** R&D Clinical Development Department, Pasteur Mérieux Connaught, Lyon, France. jlang@fr.pmc-vacc.com

## Abstract

The development of vaccines against rare emerging infectious diseases is hampered by many disincentives. In the face of growing in-house expenditures associated with research and development projects in a complex legal and regulatory environment, most pharmaceutical companies prioritize their projects and streamline their product portfolio. Nevertheless, for humanitarian reasons, there is a need to develop niche vaccines for rare diseases not preventable or curable by other means. The U.S. Orphan Drug Act of 1983 and a similar proposal from the European Commission (currently under legislative approval) provide financial and practical incentives for the research and development of drugs to treat rare diseases. In addition, updated epidemiologic information from experts in the field of emerging diseases; increased disease awareness among health professionals, patients, and the general public; a list of priority vaccines; emergence of a dedicated organization with strong leadership; and the long-term pharmacoeconomic viability of orphan products will be key factors in overcoming the complexity of orphan status and the limited need for vaccine.


### Development of Orphan Vaccines: An Industry Perspective

**Jean Lang and Susan C. Wood**
Pasteur Mérieux Connaught, Lyon, France

The development of vaccines against rare emerging infectious diseases is hampered by many disincentives. In the face of growing in-house expenditures associated with research and development projects in a complex legal and regulatory environment, most pharmaceutical companies prioritize their projects and streamline their product portfolio. Nevertheless, for humanitarian reasons, there is a need to develop niche vaccines for rare diseases not preventable or curable by other means. The U.S. Orphan Drug Act of 1983 and a similar proposal from the European Commission (currently under legislative approval) provide financial and practical incentives for the research and development of drugs to treat rare diseases. In addition, updated epidemiologic information from experts in the field of emerging diseases; increased disease awareness among health professionals, patients, and the general public; a list of priority vaccines; emergence of a dedicated organization with strong leadership; and the long-term pharmacoeconomic viability of orphan products will be key factors in overcoming the complexity of orphan status and the limited need for vaccine.

### The Problem

In considering the development of a new vaccine, preventive immunization, generally considered the most cost-effective health intervention, should be ranked against other strategies for disease control, such as case management (treatment of disease) or control of environmental factors linked to vector prevalence and dynamics (e.g., overpopulation, rural-to-urban migration, economic status, vector control, inadequate domestic water supply or sewage disposal) (1). Evaluating vaccination options and economic impact is particularly important for vaccines against low-prevalence or geographically contained emerging infectious diseases with limited demand, for which development costs may not be recovered. Thus, consensus should be reached on the mid- to long-term public health significance (e.g., vector dynamics and potential control, age prevalence and targets, risk categories, case-fatality rates, and possible future epidemiologic scenarios) of any vaccine-preventable disease. Without clear premises and long-term commitment, the development of vaccines for rare infectious diseases or those of narrow scope (e.g., geographically limited but regionally important diseases such as arboviral or diarrheal diseases) or for which development costs offset the market potential, called here orphan vaccines, may be considered a precarious venture that most organizations would hesitate to pursue.

Address for correspondence: Jean Lang, Vector-Borne Diseases Clinical Program, R&D Clinical Development Department, Pasteur Mérieux Connaught, Lyon, France; fax: 33-47-273-7928; e-mail: jlang@fr.pmc-vacc.com.

### Disincentives for Orphan Vaccine Development

### Competing Costs

Vaccine development involves a substantial investment in time, effort, and resources. Any private- or public-sector vaccine research and development process involves choices concerning the allocation of resources at all levels, including personnel and management. The costs from research to licensure, the risks inherent in vaccine development (e.g., technological constraints, regulatory approval) and the short- and long-term market financial evaluations (e.g., net present value, return on investment [2]) are key factors in the decision to develop a vaccine against a rare disease. In addition, long-term market evaluation and return on investment are often difficult to estimate because of the unpredictable nature of disease outbreaks and vector dynamics. Growing in-house expenditures associated with research and development projects in a complex legal and regulatory environment prompt most pharmaceutical companies to prioritize their projects and streamline their product portfolio (3). The same is true in the public health sector where the appearance of an orphan vaccine would increase the already tough competition for resources, as evidenced by the present shortcomings in developing countries' use of current and candidate Expanded Program of Immunization (EPI) vaccines (hepatitis B, measles, yellow fever, *Haemophilus influenzae* type b).

### Vaccine Pricing

It has been repeatedly shown that one of the most accurate predictors of the successful use of an EPI vaccine, such as hepatitis B, is not necessarily the endemicity of the disease but instead the vaccine cost per dose (4,5). Thus, the research, development, production, marketing, and distribution of a safe and effective vaccine should be assessed to determine if its potential cost per dose would be acceptable in an already difficult marketplace (4). The limited economic prospects and size of the market, with probably no prospect for economies of scale in production, are particularly relevant in the vaccine industry. Economic models of vaccine production have shown an inverse relationship between the number of doses produced and the cost per dose (6). As a consequence, a tiered pricing strategy has been endorsed by the World Health Organization (WHO), in which high-cost but low-volume vaccine sales in industrialized countries could subsidize the low cost and larger volume of sales in developing countries, although this may not be feasible if the quantity of vaccines needed in developing countries is low (6,7).

### Patent Protection and Product Liability

Introduction of new vaccines relies heavily on the strategic use of intellectual property rights to reassure investors that a candidate vaccine will provide a fair return on invested funds. The lack of patent protection or legal framework for intellectual property rights in some developing countries interferes with the long-term viability of a vaccine. In Western countries, liability issues associated with a candidate vaccine and its intended population (8) also affect development costs.

### Orphan Drugs and Vaccines Situation in the United States

The United States was the first nation to propose a legal framework to overcome the disincentives to developing orphan drugs and encourage their development and availability (9,10). The Preamble on Orphan Drugs to the legislation passed by the U.S. Congress contained the following points: 1) Many diseases and conditions (so-called orphan diseases) exist that affect very small numbers of persons; however, the overall group of patients affected by such diseases totals 20 million or more in the United States; 2) adequate drugs for orphan diseases have not been developed; 3) pharmaceutical companies may reasonably expect to generate relatively small sales in comparison to the cost of developing an orphan product; and 4) costs of developing such drugs should be reduced, and financial incentives should be provided.

The legislation defines two classes of orphan diseases. The first class comprises diseases that affect fewer than 200,000 Americans. In this case, sales of a drug, vaccine, diagnostic test, or blood product intended for use in such a disorder would be insufficient to offset the costs incurred during development and marketing of the product. This program is directed at public health needs beyond the U.S. borders, providing a stimulating factor for the U.S. pharmaceutical community to develop products to meet the needs of populations elsewhere. The second class of orphan diseases affects more than 200,000 Americans but has no potential recovery costs from U.S. sales. Thus, the program may also apply to specific subpopulations of patients with a more common disease for which the sponsor does not expect to offset development and marketing costs in the first 7 years of sales.

Concerning vaccines, the U.S. Food and Drug Administration (FDA) stipulated that, when establishing the claim for orphan status, the intended population should reflect the number of persons who would receive the vaccine annually as of the date of designation.

### Orphan Drug Incentives

To further encourage orphan drug availability, accompanying market-oriented incentives for orphan drug development were issued by the Office of Orphan Products Development, under the auspices of FDA (11). The sponsor makes the request for orphan drug status (before filing a New Drug Application or a Product License Application) on the basis of information and circumstances at the time the request is submitted.

Funds for research through Orphan Products Grants Programs benefit from a 50% deduction tax credit for clinical trial expenses (9) and a market exclusivity of 7 years. Protocol assistance in the form of written recommendations from the secretary of the Department of Health and Human Services for the nonclinical and clinical investigations needed for marketing approval are provided to accelerate the approval process. In this respect, a flexible approach has been adopted for the development of orphan drugs. For example, the preclinical dossier (i.e., the pharmaceutical and pharmacotoxologic data included in the registration file) may not have to include data on animal toxicity, and teratogenicity or carcinogenicity results may be waived in some cases (12). This flexibility in the registration requirements can be applied in certain cases to expedite the approval process but cannot be used in instances where it could compromise the safety of the consumer.

The legislation states that the clinical dossier of an orphan drug or vaccine should be built on a realistic assessment of the qualitative and quantitative nature of the studies that can be performed. This measure is relevant because the orphan nature of the disease and its prevalence in regions with limited medical facilities and services may make it difficult to recruit a large enough number of qualified participants for a clinical trial. On the other hand, the drawback of basing a clinical dossier on a limited amount of data is the obvious difficulty in evaluating the safety profile of an orphan product with sufficient statistical confidence. On average, orphan drugs may be associated with greater hazard than other products. For example, during clinical testing, 31% of orphan drugs on the market had more pronounced adverse effects than nonorphan medicinal products (13). Likewise, after FDA approval, 13% of orphan products provoked more side effects than anticipated.

To encourage development of novel orphan compounds, FDA stipulated that two products would be considered the same (and thus the latter one would not qualify for the incentives in the Orphan Drug Act), unless the second product was shown to be clinically superior to the first. This stipulation provides a clear incentive for the original manufacturer of a product likely to be reproduced, who funds the full costs of research and development. For example, in the case of two live, attenuated viral vaccines, only the first would be granted orphan status for a given preventive indication, unless the second vaccine proved clinically superior.

### Liability Coverage

Although not definitely clarified, proposals have been made to solve some specific liability issues, including design defects, duty to warn, negligence in testing or manufacturing, and defining responsibility for no-fault injury (13). The National Vaccine Compensation Program (issued in 1986), which provides no-fault compensation for vaccine-related injuries, is financed by a trust fund created by an excise tax on every dose of vaccine sold (14).

### Orphan Drugs and Vaccines in Europe

In 1994, the European Commission (the legislative body of the European Union [EU]) stated its interest in orphan diseases. In 1998, with close collaboration of the French Ministry of Health (15) and the European Medicine Evaluation Agency, a text was approved recommending the creation of a European Office for Orphan Drugs along the same lines as the U.S. Office of Orphan Products Development.

The proposed European criteria for classification of a drug as an orphan drug (including vaccines) are almost identical to the U.S. criteria, except that they are based on a disease prevalence of 5 per 10,000 Europeans (falling between the United States [7 per 10,000] and Japan [2.5 per 10,000]), when no current methods of diagnosis, prevention or treatment, or major contribution to current patient care exist.

The legislation will provide incentives to the European pharmaceutical industry in terms of research and development assistance (protocol assistance, normal evaluation, possible form of centralized but fast-track approval procedures), fee waiver, tax credits, funds from the European Orphan Product Grant Program, and market exclusivity for 10 years (interim period 6 years) and will encourage national policies (subsidiary principle, e.g., the French compassionate use authorization [Autorisation Temporaire Utilisation]) (16).

The role of patient groups in increasing awareness of orphan drug development has been widely recognized for pharmaceutical orphan drugs in the United States and has been emphasized in the European project. The potential end-users of an orphan product may not be aware of therapeutic or preventive options. The European Office of Orphan Products Development will therefore support the establishment of groups of persons with the same rare conditions to play a role in increasing awareness of the disease within the population and will coordinate their activities at national and community levels. It remains to be seen how this initiative will apply to vaccine-preventable infectious diseases in communities where individuals or groups may not be aware of the risk for infection and thus the value of the vaccine.

To clarify the extent of patent protection and the right to benefit from the orphan incentive package, the European Commission (DG24 committee) recently defined "similarity" between orphan products as the same substance, or a substance that differs from the original substance in molecular structure, source material, or manufacturing process, or an organism (living or nonliving) that is comparable with the original substance or organism in terms of biologic action and properties (including efficacy and safety) and ability to act through the same mechanism. In the same way as the U.S. legislation, this would favor the development of novel orphan products by the innovative company.

In June 1999, the European Parliament's committee on the environment, public health, and consumer protection adopted a report by one of its senior members in favor of the Policy on Orphan Drugs and proposed some amendments to widen the scope of the legislation. Among other changes, the committee requested more flexibility in the proposed provisions for clinical trials, allowing (under specific conditions) availability of the product before final authorization is granted. The committee proposed extending the definition of orphan drug status to cover products intended for serious and chronic diseases. It also recommended, as in the United States, additional incentives for developing medicinal or biologic products for diseases that occur mainly in tropical regions but rarely within EU territory. Finally, the committee called for an Orphan Medicinal Product Innovation Promotion Fund to be financed from the sales of orphan drugs after the proposed 10-year period of market exclusivity. The European Orphan Drug Policy could be enacted early in the year 2000.

### Orphan Drugs in Other Industrialized Countries

After the U.S. Orphan Drug Act, similar legislation was enacted in Japan in 1993. An Australian orphan drugs program based on the U.S. program began in 1998 (17). Since then, the Therapeutic Goods Administration has designated two biological drugs as orphans—rabies immunoglobulin and recombinant enzyme imiglucerase for replacement therapy in patients with Gaucher disease. A cross-national comparison of orphan drug and vaccine policies has been made for different countries, including Japan, Canada, France, Sweden, and the United Kingdom (12).

### Orphan Vaccines in Developing Countries

The availability and use of orphan vaccines in developing countries are complex since these countries have yet to ensure optimum use of existing priority vaccines (17). The limitations and obstacles involved in expanding the use of these priority vaccines are further multiplied for orphan vaccines of limited need. Within the framework of WHO, the Children's Vaccine Initiative set the development of vaccines with commercial prospects as a priority measure (7). This cost-oriented definition reflects mainly the difficulty of developing vaccines and drugs for tropical diseases, even those as prevalent as malaria (19,20). The Children's Vaccine Initiative's role has been problematic for various reasons (21), and this structure has faced increasing difficulties in maintaining its visibility.

Nevertheless, other noneconomic factors (3) could justify an industry's decision to develop and market an orphan vaccine: desire to enhance the company's ethical profile by fulfilling a medical or social need; capacity to develop, produce, and market the drug; a larger company strategy (e.g., part of a product range); and possible additional uses that would increase the drug's future economic viability. The latter point may be less relevant for vaccines, which are usually tailor-made to their infectious agents.

In the development of any vaccine against an emerging infectious disease, certain general rules apply (4,6,18,22), for example, developing strong research and development capacity, obtaining reliable scientific results and training in industrialized and developing countries; bulk filling arrangements; licensing technology; negotiating partnerships for specific products; joint venture agreements with western research and development manufacturers (economic value of the alliance); identifying the neediest countries on the basis of a banding strategy that classes countries according to their gross national product per capita, thereby allowing tiered pricing among them (6,23); and creation of funding mechanisms. Some could argue that from an industry perspective, if all of these criteria cannot be met, the vaccine should not be developed.

No trade-off on the quality of an orphan vaccine is ethically justified or accepted. For the pharmaceutical industry, therefore, the costs incurred in development, ensuring tight quality controls, and establishing industrial good manufacturing procedures for an orphan vaccine are similar to those incurred with a traditional vaccine. For this reason, the development of any orphan vaccine should be broadly supported by measures to increase the awareness of immunization benefits at three levels—the decision-makers, the caregivers, and the patients.

### Increasing Orphan Vaccine Availability

Development of orphan vaccines is guided by the limited need for or market potential of the product, with the accompanying regulations, as well as the specific characteristics of the vaccine and those who need it (24). Because of the pitfalls related to these limitations, few orphan vaccines have reached the neediest populations. For example, in the United States, by the end of 1997, 837 medicinal products had been designated orphan drugs; 152 of these obtained authorization. This number was a clear improvement over that of the previous 14 years, during which 34 medicinal orphan products obtained authorization. However, our website review found only eight vaccines registered with orphan status (seven for therapeutic indications [e.g., cancer and sickle cell anemia], one to prevent an Asiatic infectious disease—Japanese encephalitis virus) and, to our knowledge, none has yet obtained final authorization. In addition, "It is not vaccines that save lives but vaccination." Even when orphan vaccines are available, we have to examine the feasibility of getting them to the intended population.

Various strategies, proposals, and recommendations for overcoming limitations inherent in orphan vaccine development and availability are listed in the Table.

### Providing Information, Prioritizing, and Securing Demand

Although funding is a major obstacle to orphan vaccine development, it may not be the only impediment to the introduction of new vaccines (25). Reliable information on the epidemiology, disease severity, and effect on public health is essential to substantiating the need for a vaccine and may not be available to support the development decision. Market forces may not always be good cultivators of vaccines, which, unlike some chemical drugs, are not big money-making products. For this reason, the public and decision makers should know about the benefits of immunization, to increase disease awareness, and support an orphan vaccine initiative.

### Facilitating Vaccine Research and Development and National and Regional Approvals

An accelerated procedure for final authorization and exemptions from all or part of the registration fee can reduce development costs, staffing requirements, and time to market and render the development of an orphan vaccine more attractive for the sponsor. Local initiatives may also speed the authorization process.

### Ensuring Market and Funding Visibility, Production, and Distribution

Finally, increasing patent protection and the defined period of market exclusivity reduces investment risks for manufacturers. Furthermore, funding for orphan projects may be advanced from private bodies looking to capitalize on an ethical business image. Indeed, the private sector looks to take on an increasingly important role in international health development, especially in poorer countries (26). Increased world travel and the risk for transport of pathogens across borders (27) support tiered pricing between the western traveler and the disease-endemic country. In addition, an orphan infectious agent in a remote developing country requiring an orphan vaccine with limited need could, over time, become an emerging disease worldwide. HIV is a case in point: a disease originating in Africa that has successfully spread to the industrialized world.

In Argentina, strong political and governmental support, aided by the U.S. Army Medical Research Institute of Infectious Diseases collaboration, ultimately culminated in a successful Candid 1 vaccination campaign against Argentinean hemorrhagic fever in agricultural workers (28). Close collaboration between the pharmaceutical sector, WHO, and the Chinese government resulted in the development of the antimalarial drug artemisin (29).

Table. Solutions and proposals for accelerating orphan vaccine availability

1. Provide information, prioritize, and secure demand
   - Increase awareness of disease: set-up of special interest groups (patients, parents, professionals), expert groups, and national forums.
   - Acquire epidemiologic data on selected infectious diseases to guide decision-making: obtain access to data registries with comparable case-definitions across countries, and obtain information from specialized units and experts, scientific literature, patient organizations,and pharmaceutical manufacturers associations.
   - Establish the suitability of vaccine prevention vs. other options: realistic comparisons of vaccination with patterns and costs of other alternatives, such as treatment or vector control.
   - Ensure political support for orphan vaccine initiatives and organize tripartite partnerships between public, private, and nongovernmental sectors.

2. Facilitate vaccine research and development and national/regional approvals
   - Promote innovative research and development technologies that could be applied to blockbuster vaccines or, alternatively, promote low-cost traditional vaccine technologies.
   - Encourage public/private sector links: academic/industrial research groups.
   - Set international standards of quality, safety, and efficacy and define minimum amount of data required for licensure.
   - Make recommendations on appropriate schedules, target ages.
   - Promote national and regional ex-U.S. and European Community incentives on Orphan Drug Policies (Latin America, Asia).
   - Expand and harmonize orphan drug policies as part of the ICH process (decrease time to regulatory approval).

3. Ensure market/funding visibility, production and distribution
   - Reduce investment risks for manufacturers by providing realistic demand estimates.
   - Fund development of orphan vaccines for developing countries through various institutional bodies, such as CVI, WHO, UNICEF, PAHO, WB, USAID, NIH, CDC, PATH, other donor bodies, and nongovernmental organizations and foundations (e.g., Gates Foundation) on the basis of target assistance for the neediest countries based on total gross national product.
   - Strengthen political and public health collaboration between orphan programs (European Community, United States) and other countries to create a supranational office dedicated to orphan vaccines (World Office of Orphan Vaccine Development or CVI) that could harmonize and coordinate funding (from research to manufacturing) from various sources.
   - Identify and expand the pool of the committed purchasers based on expected coverage criteria.
   - Promote and support protection of intellectual property.
   - Clarify compensation programs that may assume responsibility for liability.
   - Evaluate tiered pricing (high/low) feasibility at two levels:
     - Multinational: traveler or military vaccines in industrialized countries, endemic community vaccines in developing countries.
     - National: a private market for the high GNP per capita subgroup, a public market for the low GNP per capita subgroup.
   - Establish manufacturing strategies, such as campaigning to subsidize orphan vaccine cost investments by large volume sales of EPI vaccines.
   - Strengthen the vaccine distribution network for the targeted population.

ICH, International Conference on Harmonization; CVI, Children's Vaccine Initiative; WHO, World Health Organization; UNICEF, United Nation's Children's Fund; PAHO, PanAmerican Health Organization; WB, World Bank; USAID, U.S. Agency for International Development; NIH, National Institutes of Health; CDC, Centers for Disease Control and Prevention; PATH, Program for Appropriate Technology in Health; EPI, Expanded Program of Immunization.

### Conclusions

The dilemmas intrinsic to the development and distribution of orphan vaccines against emerging infectious diseases reflect many of the issues faced by policy makers worldwide with regards to cost, quality of care, access to care, and the role of government intervention in regulating the health-care market (30). In view of the current globalization of trades and markets, worldwide orphan vaccine policies and a specialized organization with a strong leadership and commitment similar to the Children's Vaccine Initiative project for a National Vaccine Authority may be needed (6,18). This kind of organization could be responsible for establishing a list of priority orphan vaccines and indicating reasons for not including other vaccines. The organization could also oversee all stages of vaccine development and have access to funds that could rapidly be mobilized. Such a global structure could serve as a forum for discussing the current limitations on orphan vaccine development and availability. Nevertheless, the problems recently faced by the Children's Vaccine Initiative indicate the difficulties in mounting and maintaining such a worldwide initiative.

### Acknowledgments

The authors thank Stanley Plotkin and the two reviewers whose constructive criticism and advice helped to improve this manuscript.

Dr. Lang is a researcher at Pasteur Mérieux Connaught, the vaccine manufacturer. His clinical research interests include rabies, dengue, Japanese encephalitis, yellow fever, malaria, and antivenoms as part of the Vector Borne Vaccine Clinical Research and Development Program.

Dr. Wood is responsible for education in vaccinology and vaccine policy for the international business unit of Pasteur Mérieux Connaught, which covers all countries outside Western Europe and North America.

### References

1. Dodet B, Saluzzo J. Factor in the emergence of arbovirus diseases. (Les Pensières): Veyrier du Lac Elsevier Science 1997; 8-10 Decembre 1996.
2. Brealey RA, Myers SC. In: Principles of corporate finance. 3rd ed. Singapore: McGraw-Hill; 1988.
3. Stucki J. The development of orphan drugs—a pharmaceutical company perspective. Cooperative approaches to research and development of orphan drugs 1985;95-104.
4. Aylward B, Kane M, Batson A, Scott R. A framework for the evaluation of vaccines for use in expanded programme on immunization. Vaccine 1994;12:1155-9.
5. Shepard DS, Walsh JA, Kleinau E, Stansfield S, Bhalotra S. Setting priorities for the Children's Vaccine Initiative: a cost-effectiveness approach. Vaccine 1995;13:707-14.
6. Milstien J, Batson A. Accelerating availability of new vaccines: the role of the international community. Drug Inf J 1998;32:175-82.
7. CVI (Children's vaccine initiative). The CVI strategic plan—managing opportunity and change: a vision of vaccination for the 21st century. OMS 1997;CVI/GEN(97-04):1-92.
8. Trannoy E. Will ethical and liability issues and public acceptance allow maternal immunization? Vaccine 1998;16:1482-5.
9. Haffner M. Orphan products: origins, progress, and prospects. Annu Rev Pharmacol Toxicol 1991;31:603-20.
10. Haffner M, Kelsey J. Evaluation of orphan products by the U.S. Food and Drug Administration. Int J Tech Assess Health Care 1992;8:647-57.
11. Cramer R. Orphan drugs improved medical treatment of rare diseases. Alabama J Med Sci 1998;25:257-67.
12. Thamer M, Brennan N, Semansky R. A cross-national comparison of orphan drug policies: implications for the U.S. Orphan Drug Act. J Health Pol Policy Law 1998;23:265-90.
13. Scharf S. Orphan drugs: the question of product liability. Am J Law Med 1985;10:491-513.
14. Bloom B. The United States needs a national vaccine authority. Science 1994;265:1378-80.
15. Brandissou S, Yagoubi N, Hasselot N. Les médicaments orphelins: problème de santé publique et enjeux économiques. Thérapie 1996;51:647-53.
16. Benzi G, Ceci A. Drugs trying to get the patents: there will be incentives for the European scientific community to develop research in the field of the orphan drugs. Pharmacol Res 1997;35:89-93.
17. Hillcoat BL. Rare diseases and "orphan" drugs [editorial]. Med J Aust 1998;169:69-70.
18. Mahoney RT, Maynard JE. The introduction of new vaccines into developing countries. Vaccine 1999;17:646-52.
19. Local initiative aids in fight against malaria [editorial]. Lancet 1998;352:212.
20. Ollario P. Will the fight against tropical diseases benefit from orphan drug status? Trop Med Int Health 1997;2:113-5.
21. Muraskin W. The politics of international health: the children's vaccine initiative and the struggle to develop vaccines for the third world. Albany (NY): State University of New York Press; 1998.
22. Milstien J, Batson A, Meaney W. A systematic method for evaluation of the potential viability of local vaccine producers. Vaccine 1997;15:1358-63.
23. Batson A. Sustainable introduction of affordable new vaccines: the targeting strategy. Vaccine 1998;16:S93-8.
24. Bloom B. Bumps on the vaccine road. Science 1994;265:1371-3.
25. Hausdorff WP. Prospects for the use of new vaccines in developing countries: cost is not the only impediment. Vaccine 1996;14:1179-86.

26. Public-private partnerships—business as usual [editorial]. Lancet 1998;352:212.
27. Spier R. Report on a meeting of a working party sponsored by the European Commission to discuss the ethical, legal and social aspects of research on vaccines and vaccination. Vaccine 1999;17:400-2.
28. Maiztegui J, MacKee K, Barrera Oro J, Harrison L, Gibbs P, Feuillade M, et al. Protective efficacy of a live attenuated vaccine against Argentine hemorrhagic fever. J Infect Dis 1998;177:277-83.
29. Helenport J, Roche G. What have been the strategies for the registration, positioning and control of medical information for im artemether? The rational use of Qinghaosu and its derivatives. 1998; Les Pensières (19-22 April):141-7, Fondation Merieux Eds.
30. Beutels P. Economic evaluations applied to HB vaccination: general observations. Vaccine 1998;16:S84-92.

## References

[PDF_00688] Beutels P. (1998). Economic evaluations applied to HB vaccination: general observations.. Vaccine.

[PDF_00668] Hausdorff W. P. (1996). Prospects for the use of new vaccines in developing countries: cost is not the only impediment.. Vaccine.

[PDF_00647] Hillcoat B. L. (1998). Rare diseases and "orphan" drugs.. Med J Aust.

[PDF_00649] Mahoney R. T., Maynard J. E. (1999). The introduction of new vaccines into developing countries.. Vaccine.

[PDF_00679] Maiztegui J. I., McKee K. T., Barrera Oro J. G., Harrison L. H., Gibbs P. H., Feuillade M. R., Enria D. A., Briggiler A. M., Levis S. C., Ambrosio A. M. (1998). Protective efficacy of a live attenuated vaccine against Argentine hemorrhagic fever. AHF Study Group.. J Infect Dis.

[PDF_00636] Scharf S. F. (1985). Orphan drugs: the question of products liability.. Am J Law Med.

[PDF_00611] Shepard D. S., Walsh J. A., Kleinau E., Stansfield S., Bhalotra S. (1995). Setting priorities for the Children's Vaccine Initiative: a cost-effectiveness approach.. Vaccine.

[PDF_00632] Thamer M., Brennan N., Semansky R. (1998). A cross-national comparison of orphan drug policies: implications for the U.S. Orphan Drug Act.. J Health Polit Policy Law.

[PDF_00622] Trannoy E. (1998). Will ethical and liability issues and public acceptance allow maternal immunization?. Vaccine.

